# Supplementary material for: Evaluation of Anticancer and Anti-Inflammatory Activities of Some Synthetic Rearranged Abietanes
Source: Int J Mol Sci. 2023 Sep 1;24(17):13583. doi: 10.3390/ijms241713583 (PMC10487843; doi:10.3390/ijms241713583)
Supplement: Supplementary file 1 [file ijms-24-13583-s001.zip › ijms-2562374-supplementary.pdf]

Supplementary Materials

Evaluation of Anticancer and Anti-Inflammatory Activities of Some Synthetic Rearranged Abietanes

Mustapha Ait El Had [1,†], Houda Zentar [1,2,†], Blanca Ruiz-Muñoz [2], Juan Sainz [2,3], Juan J. Guardia [1], Antonio Fernández [1], José Justicia [1], Enrique Alvarez-Manzaneda [1], Fernando J. Reyes-Zurita [2,*] and Rachid Chahboun [1,*]

1 Departamento de Química Orgánica, Facultad de Ciencias, Instituto de Biotecnología, Universidad de Granada, 18071 Granada, Spain
2 Departamento de Bioquímica y Biología Molecular I, Facultad de Ciencias, Universidad de Granada, 18071 Granada, Spain
3 Centre for Genomics and Oncological Research: Pfizer, Genomic Oncology Area, GENYO, University of Granada, Andalusian Regional Government, PTS Granada, 18016 Granada, Spain
* Correspondence: ferjes@ugr.es (F.J.R.-Z.); rachid@ugr.es (R.C.); Fax: +34-958-248-437 (R.C.)
† These authors contributed equally to this work.

	page
General Procedures	S2
Experimental Procedures	S2-S6
^{1}H and ^{13}C NMR spectra of compounds	S7-S28

General Procedures

Unless stated otherwise, reactions were performed in oven-dried glassware under an argon atmosphere using dry solvents. Solvents were dried as follows: dichloromethane (DCM), Tetrahydrofuran (THF) and Dioxane. Thin-layer chromatography (TLC) was performed using F254 precoated plates (0.25 mm) and visualized by UV fluorescence quenching and phosphomolybdic acid solution staining. Flash chromatography was performed on silica gel (230-400 mesh). Chromatography separations were carried out by conventional column on silica gel 60 (230-400 Mesh), using hexanes-AcOEt (AcOEt-hexane) mixtures of increasing polarity. 1H and ^{13}C NMR spectra were recorded at 500 and 125 MHz, respectively. $CDCl_3$ was treated with K_2CO_3. Chemical shifts (δH) are quoted in parts per million (ppm) referenced to the appropriate residual solvent peak and tetramethylsilane. Data for 1H NMR spectra are reported as follows: chemical shift (δ ppm) (multiplicity, coupling constant (Hz), integration), with the abbreviations s, br s, d, br d, t, q, hept and m denoting singlet, broad singlet, doublet, broad doublet, triplet, quartet, heptuplete and multiplet, respectively. *J* = coupling constant in Hertz (Hz). Data for ^{13}C NMR spectra are reported in terms of chemical shift relative to Me_4Si (δ 0.0) and the signals were assigned utilizing DEPT experiments and on the basis of heteronuclear correlations. Infrared spectra (IR) were recorded on a FTIR spectrophotometer and are reported in frequency of absorption (cm^{-1}). Only selected absorbances (ν max) are reported.

Experimental Procedures

Synthesis of saprorthoquinone (7) from catechol 18

To a solution of catechol 18 (156 mg, 0.52 mmol) in dry 1,4-dioxane (7 mL) were added SeO_2 (64 mg, 0.57 mmol) and two drops of conc.H_2SO_4. The resulting mixture was refluxed for 10 min at which time TLC showed no starting material. Then, the solvent was evaporated and the resulting crude product was diluted with AcOEt-water (20 : 5 mL), the phases were shaken and separated. The organic phase was washed with water (2 x 5 mL), brine (5 mL) dried over anh. Na_2SO_4, filtered and evaporated to give a crude product, which was purified by flash column chromatography on silica gel, using 5% EtOAc/hexanes to afford 154 mg of saprorthoquinone (**7**) (88%) as a yellow solid. ^{1}H NMR (500 MHz, Chloroform-*d*) δ 7.36 (d, *J* = 7.5 Hz, 1H), 7.08 (s, 1H), 7.05 (d, *J* =

7.5 Hz, 1H), 5.31 (t, J = 7.7 Hz, 1H), 3.14 – 3.05 (m, 2H), 3.04 (hept, J = 6.8 Hz, 1H), 2.39 (s, 3H), 2.29 – 2.14 (m, 2H), 1.71 (s, 3H), 1.63 (s, 3H), 1.18 (d, J = 6.9 Hz, 6H). ^{13}C NMR (126 MHz, Chloroform-*d*) δ 182.54 (C=O), 181.66 (C=O), 148.18 (C), 144.72 (C), 140.29(CH), 140.23 (C), 136.63(CH), 134.93 (C), 132.37 (C), 128.54 (C), 128.10 (CH), 123.82 (CH), 30.25 (CH_2), 27.54 (CH_2), 26.91 (CH), 25.74 (CH_3), 21.52 (2 × CH_3), 19.94 (CH_3), 17.63 (CH_3). IR (film): 3018, 1691, 1661, 1636, 1260, 900 cm^{-1}. HRMS (ESI) m/z: calcd for $C_{20}H_{24}O_2$ (M+H$^+$) 296.1777, found: 296.1759.

Synthesis of *ortho*-quinone 13 from ferruginol (1)

$(PhSeO)_2O$ (251 mg, 0.7 mmol) was added to a solution of phenol 1 (200 mg, 0.7 mmol) in anhydrous THF (8 mL) under an argon atmosphere, and the reaction was stirred at reflux for 15 minutes. The solvent was evaporated under vacuum to yield a crude product, which was then subjected to column chromatography on silica gel using a 4% EtOAc/hexane eluent. This procedure afforded quinone 13 (142 mg, 74%) as a green solid. ^{1}H NMR (500 MHz, Chloroform-*d*) δ 6.38 (s, 1H), 2.88 (hept, J = 6.9 Hz, 1H), 2.75 – 2.67 (m, 2H), 2.52 – 2.34 (m, 2H), 1.83 (ddt, J = 13.6, 7.0, 1.8 Hz, 1H), 1.65 (qt, J = 13.2, 3.4 Hz, 1H), 1.56 – 1.39 (m, 2H), 1.21 (s, 3H), 1.08 (d, J = 2.5 Hz, 3H), 1.07 (d, J = 2.5 Hz, 3H), 0.91 (s, 3H), 0.87 (s, 3H). ^{13}C NMR (126 MHz, $cdcl_3$) δ 181.23 (C=O), 180.24 (C=O), 147.91 (C), 146.61 (C), 144.84 (C), 137.86 (CH), 51.28 (CH), 41.53(CH_2), 38.00 (C), 36.01 (CH_2), 33.78 (CH_2), 33.45 (CH_3), 33.38 (C),, 26.87 (CH), 21.69 (CH_3), 21.41 (CH_3), 21.37 (CH_3), 19.97 (CH_3), 18.84 (CH_2), 18.05 (CH_2). IR (film): 3443, 3314, 3289, 2967, 2923, 1648, 1573, 1465, 1391, 1270, 1175, 1103, 1044, 975, 905, 755, 695, 655. HR-MS (ESI) m/z: Calcd for $C_{20}H_{28}O_2$ 300.2089, found: 300.2069.

Synthesis of saprorthoquinone (7) from *ortho*-quinone 13

To a solution of 13 (93 mg, 0.31 mmol) in dry 1,4-dioxane (5 mL) was added and conc.H_2SO_4 (40 ml, 0.74 mmol) and the reaction mixture was stirred at room temperature for 30 min, then SeO_2 (38 mg, 0.34 mmol) was added, and the mixture was refluxed for 20 min, at which time TLC showed no starting material. The reaction was quenched with water (5 mL), the solvent was evaporated, and the resulting crude product was diluted with AcOEt (15 mL). The phases were shaken and separated, the organic phase was washed with water (2 x 5 mL) and brine (5 mL). Next, it was dried over anh. Na_2SO_4, filtered and evaporated to give a crude product, which was purified

by flash column chromatography on-silica gel, using 5% EtOAc/hexanes to provide 74 mg of saprorthoquinone (7) (81%) as a yellow solid.

(E)-5-(6-isopropyl-2-methyl-7,8-dioxo-7,8-dihydronaphthalen-1-yl)-2-methylpent-2-enal (20)

To a solution of catechol 18 (112 mg, 0.37 mmol) in dry 1,4-dioxane (7 mL) were added SeO_2 (64 mg, 0.57 mmol) and conc.H_2SO_4 (19 ml, 0.34 mmol). The resulting mixture was refluxed for 15 min at which time TLC showed no starting material. Then, the reaction was quenched with water (5 mL), the solvent was evaporated, and the resulting crude product was diluted with AcOEt (15 mL). The phases were shaken and separated, the organic phase was washed with water (2 x 5 mL) and brine (5 mL). Next, it was dried over anh. Na_2SO_4, filtered and evaporated to give a crude product, which was purified by flash column chromatography on silica gel, using 5% EtOAc/hexanes to afford 75 mg of saprorthoquinone derivative 20 (65%) as a yellow syrup. ^{1}H NMR (500 MHz, Chloroform-*d*) δ 9.45 (s, 1H), 7.42 (d, *J* = 7.7 Hz, 1H), 7.11 (s, 1H), 7.10 (d, *J* = 7.7 Hz, 1H), 6.69 (t, *J* = 7.5 Hz, 1H), 3.21 (t, *J* = 10 Hz, 2H), 3.03 (hept, *J* =6.9 Hz, 1H), 2.59 (q, *J* = 7.5 Hz, 2H), 2.41 (s, 3H), 1.77 (s, 3H), 1.18 (d, *J* = 6.9 Hz, 6H). ^{13}C NMR (126 MHz, $CDCl_3$) δ 195.39 (CHO), 182.53 (C=O), 181.26 (C=O), 153.26 (CH), 146.32 (C), 145.08 (C), 140.11 (CH), 139.89 (C), 139.71 (C), 137.13 (CH), 135.27 (C), 128.69 (CH), 128.45 (C), 28.79 (CH_2), 28.22 (CH_2), 26.99 (CH), 21.50 (2 × CH_3), 19.90 (CH_3), 9.18 (CH_3). IR (film): 1723, 1701, 1667, 1640, 1636, 1260, 912 cm^{-1}. HRMS (ESI) m/z: calcd for $C_{20}H_{22}O_3$ ($M+H^+$) 310.1569, found: 310.1565.

Synthesis of 1-deoxyviroxocin (12).

To a solution of triphenylphosphine (105 mg, 0.4 mmol) in CH_2Cl_2 (5 mL) was added iodine (102 mg, 0.4 mmol) and the mixture was stirred for 5 min. Then, a solution of hydroxy enone 21 (120 mg, 0.4 mmol) in CH_2Cl_2 (5 mL) was added and the resulting mixture was stirred at room temperature for 10 min at which time TLC showed no starting material. The reaction was quenched with 10% solution of $NaHSO_3$ (5 mL), and the dark reaction mixture was stirred vigorously until its color turns yellow. Then, it was diluted with AcOEt (15 mL) and the phases were shaken and separated. the organic phase was washed with water (5 mL) and brine (5 mL). Next, it was dried over anh. Na_2SO_4, filtered and evaporated to give a crude product, which was purified by flash column chromatography on silica gel, using 10% EtOAc/hexanes to give 117 mg of 1-

deoxyviroxocin (12) (98%) as a colorless syrup. ^1H NMR (500 MHz, chloroform-d) δ (ppm): 7.48 (d, J = 8.3 Hz, 1H), 7.35 (s, 1H), 7.09 (d, J = 8.3 Hz, 1H), 6.35 (s, 1H), 3.93 (td, J = 13.7, 5.3 Hz, 1H), 3.36 (hept, J_1 = 6.9, J_2 = 0.8, 1H), 2.93 (ddd, J = 13.2, 5.5, 2.4 Hz, 1H), 2.44 (s, 3H), 2.19 (m, 1H), 1.69 (m, 1H), 1.65 (s, 3H), 1.60 (m; 1H), 1.36 (d, J = 6.9 Hz, 3H), 1.32 (d, J = 6.9 Hz, 3H), 1.24 (m, 1H), 1.16 (s, 3H). ^{13}C NMR (125 MHz, Chloroform-d) δ (ppm): 147.50 (C), 135.08 (C), 134.33 (C), 132.84 (C), 131.81 (C), 129.86 (C), 127.86 (C), 126.86 (CH), 126.02 (CH), 120.98 (CH), 85.20 (C), 33.52 (CH_2), 27.50 (CH_3), 27.86 (CH), 26.68 (CH_2), 26.38 (CH_3), 23.22 (CH_2). 22.29 (CH_3), 22.43 (CH_3), 19.82 (CH_3). IR (film): 3498, 2965, 2931, 2870, 1708, 1453, 1417, 1371, 1314, 1224, 1211, 1171, 1116, 997, 943, 878, 823, 791, 759, 677 cm^{-1}. HRMS (ESI) m/z: calcd for $C_{20}H_{27}O_2$ (M+H$^+$) 299.2006, found: 299.1991.

1-deoxyviroxocin (12) :

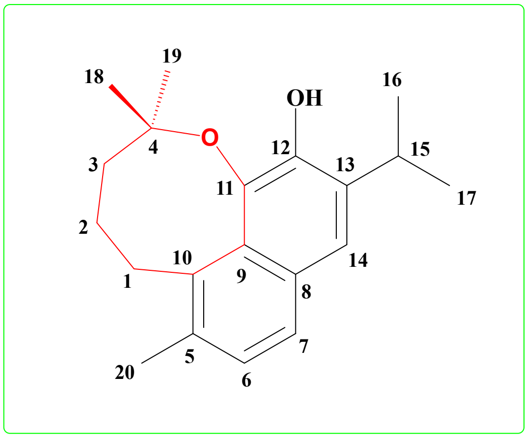

Table S1. ^{1}H NMR data for the synthetic and natural 1-deoxyviroxocin (12).

Position	Natural **1-deoxyviroxocin (12)** (Ref [17], 400 MHz)	Synthetic **1-deoxyviroxocin (12)** (500 MHz)
1	α, 2.92, ddd (J_1 = 13.2, J_2 = 5.5, J_3 = 2.3); β, 3.92, td (J_1 = 13.6, J_2 = 4.9)	α, 2.93 (ddd, J = 13.2, 5.5, 2.4 Hz, 1H); β, 3.93 (td, J = 13.7, 5.3 Hz, 1H)
2	α, 2.17, m; β, 1.67, m	α, 2.19 (m, 1H); β, 1.69 (m, 1H)
3	α, 1.60, m; β, 1.23, m	α, 1.60 (m; 1H); β, 1.24 (m, 1H)
4	-	-
5	-	-
6	7.08, d (J_1 = 8.3)	7.09 (d, J = 8.3 Hz, 1H)
7	7.47, d (J_1 = 8.3)	7.48 (d, J = 8.3 Hz, 1H)
8	-	-
9	-	-
10	-	-
11	-	-
12	-	-
13	-	-
14	7.34, br (s)	7.35 (s, 1H)
15	3.35, septd (J_1 = 6.9, J_2 = 0.8)	3.36 (septd, J_1 = 6.9, J_2 = 0.8, 1H)
16	1.31, d (J_1 = 6.9)	1.32 (d, J = 6.9 Hz, 3H)
17	1.35, d (J_1 = 6.9)	1.36 (d, J = 6.9 Hz, 3H)
18	1.15, s	1.16 (s, 3H)
19	1.64, s	1.65 (s, 3H)
20	2.43, s	2.44 (s, 3H)
12-OH	6.34, s	6.35 (s, 1H)

Table S2. ^{13}C NMR data for the synthetic and natural deoxyviroxocin (12).

Position	Natural **1-deoxyviroxocin (12)** (Ref [17], 100 MHz)	Synthetic **1-deoxyviroxocin (12)** (125 MHz)
1	26.7	26.68
2	23.2	23.22
3	33.5	33.52
4	85.2	85.20
5	132.8	132.84
6	126.8	126.86
7	126.0	126.02
8	127.9	127.86

9	129.8	129.86
10	131.8	131.81
11	134.3	134.33
12	147.5	147.50
13	135.1	135.08
14	121.0	120.98
15	28.0	27.86
16	22.5	22.29
17	22.6	22.43
18	27.7	27.50
19	26.5	26.38
20	20.0	19.82

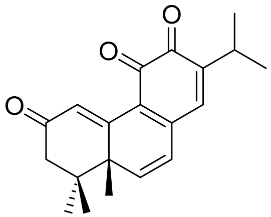

Pygmaeocin B (5)

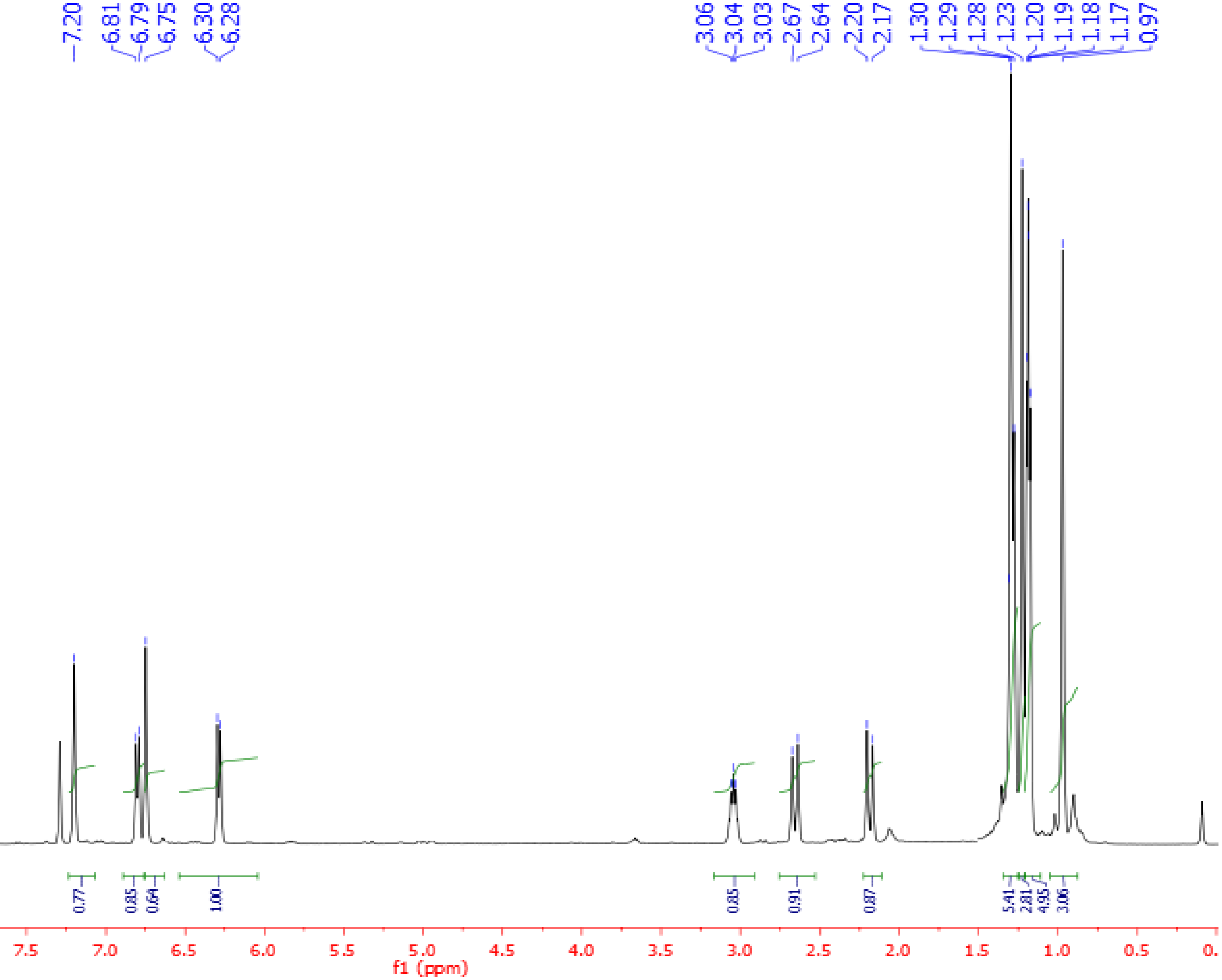

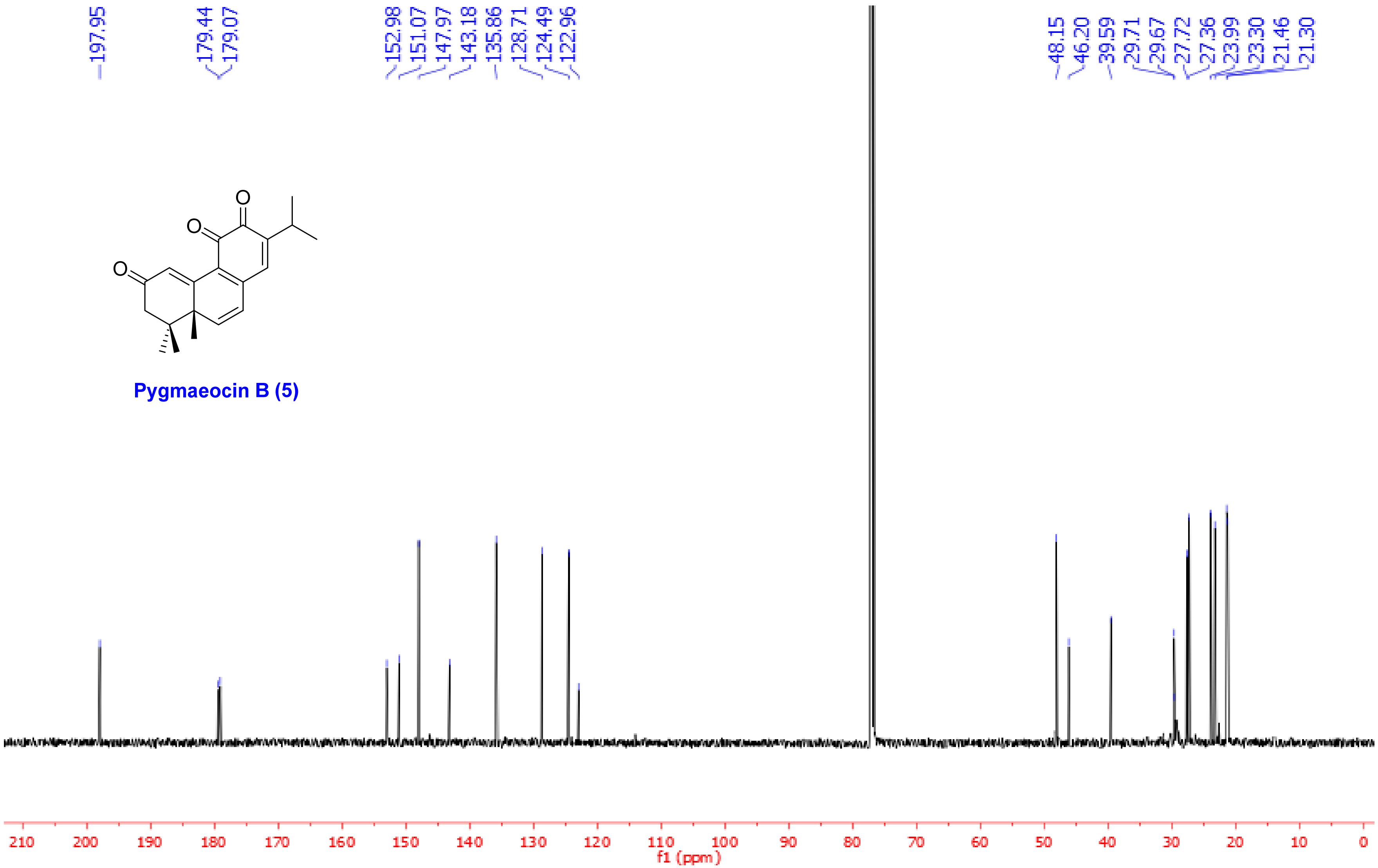

Pygmaeocin B (5)

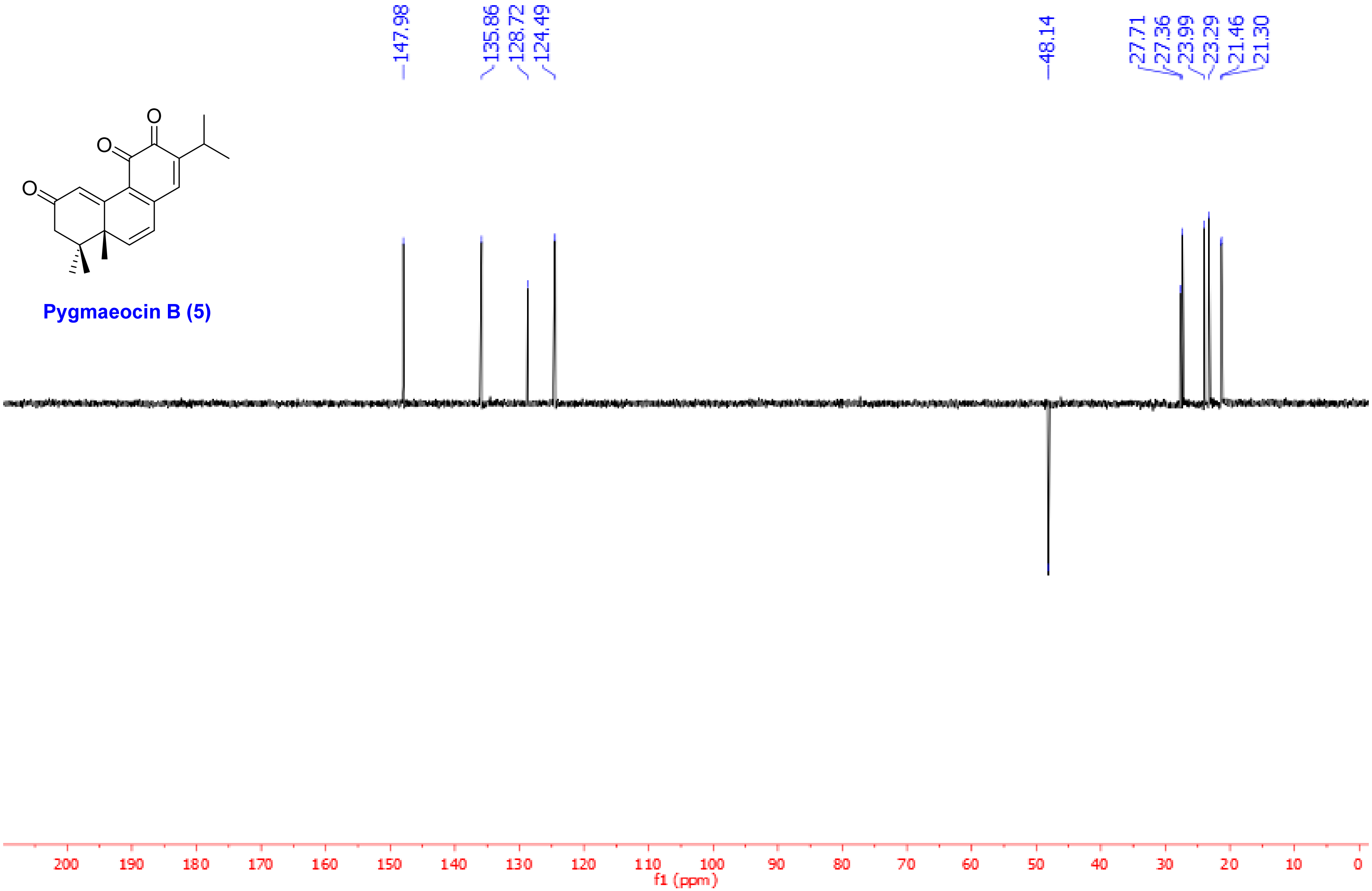
147.98
135.86
128.72
124.49
48.14
27.71
27.36
23.99
23.29
21.46
21.30
Pygmaeocin B (5)
f1 (ppm)

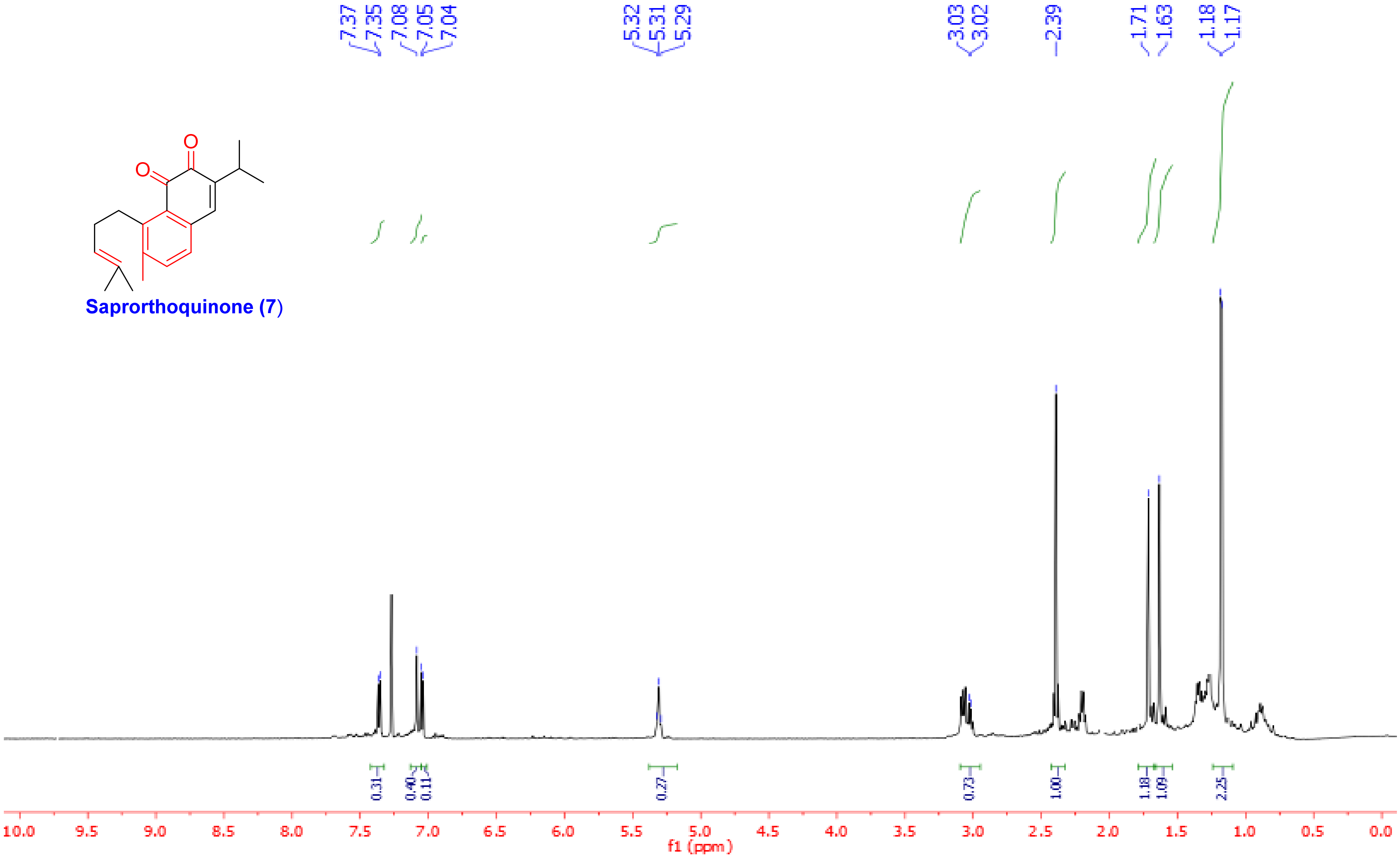
Saprorthoquinone (7)
7.37
7.35
7.08
7.05
7.04
5.32
5.31
5.29
3.03
3.02
2.39
1.71
1.63
1.18
1.17
0.31
0.40
0.11
0.27
0.73
1.00
1.18
1.09
2.25
10.0
9.5
9.0
8.5
8.0
7.5
7.0
6.5
6.0
5.5
5.0
4.5
4.0
3.5
3.0
2.5
2.0
1.5
1.0
0.5
0.0
f1 (ppm)

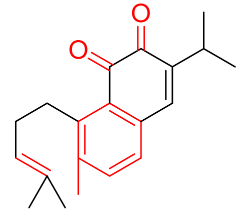

Saprorthoquinone (7)

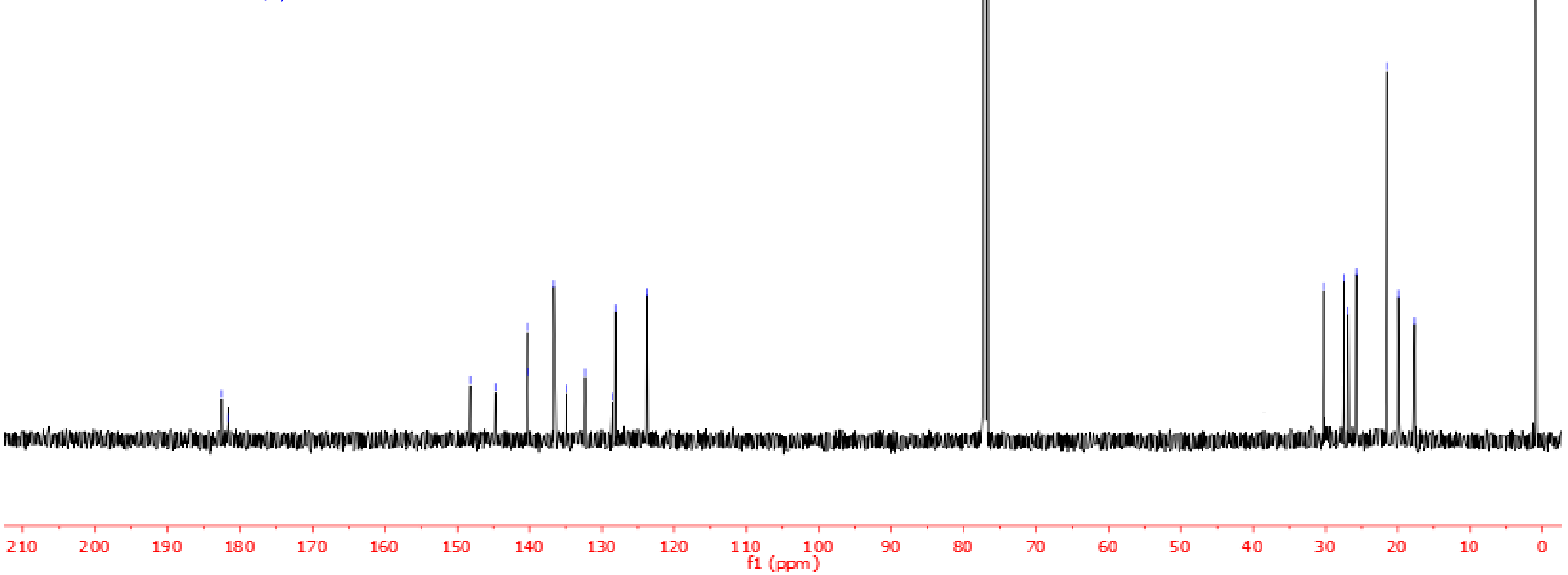

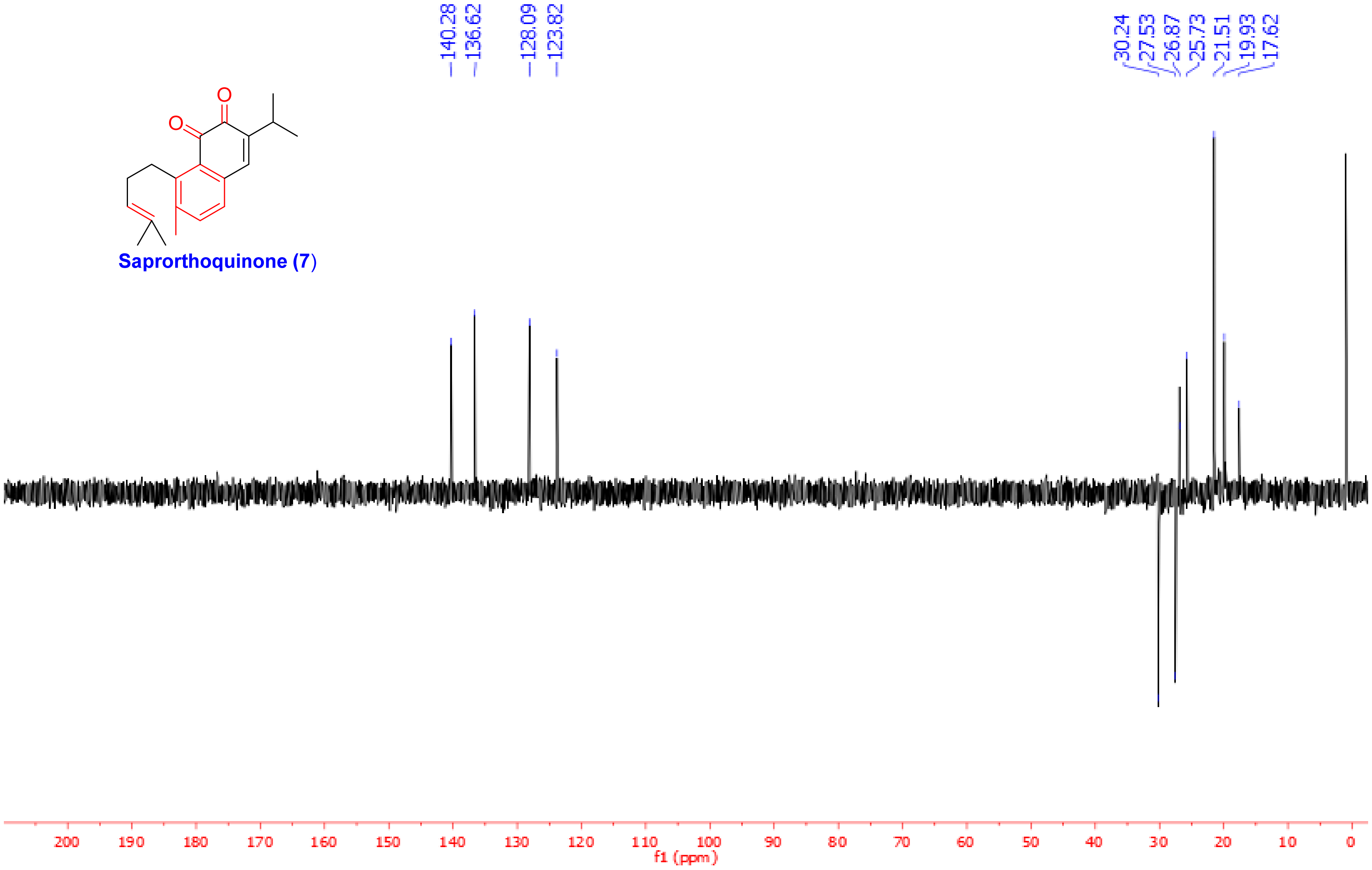
Saprorthoquinone (7)
140.28
136.62
128.09
123.82
30.24
27.53
26.87
25.73
21.51
19.93
17.62
200 190 180 170 160 150 140 130 120 110 100 90 80 70 60 50 40 30 20 10 0
f1 (ppm)

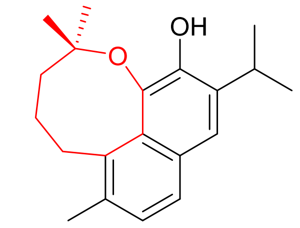

1-deoxyviroxocin (12)

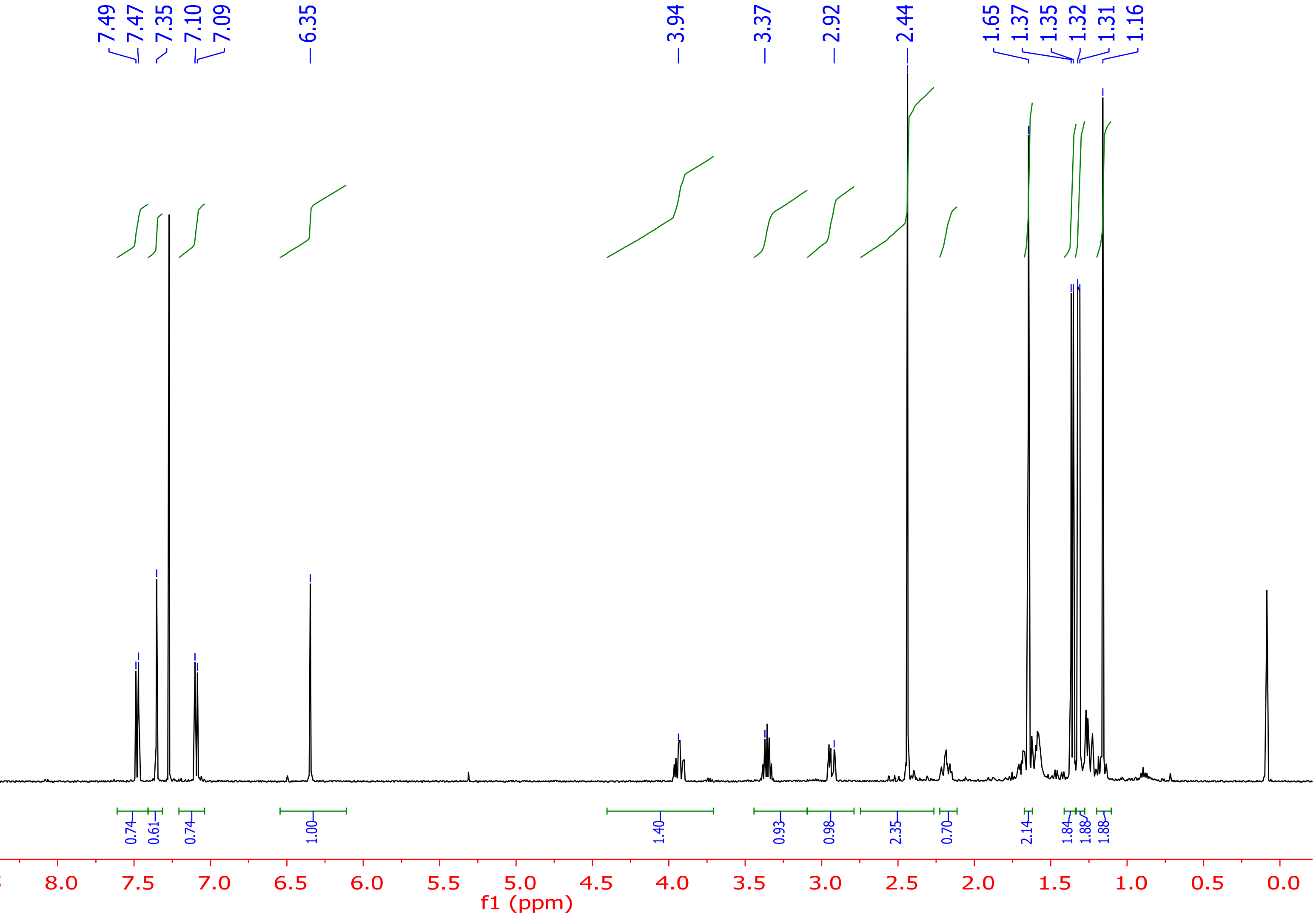

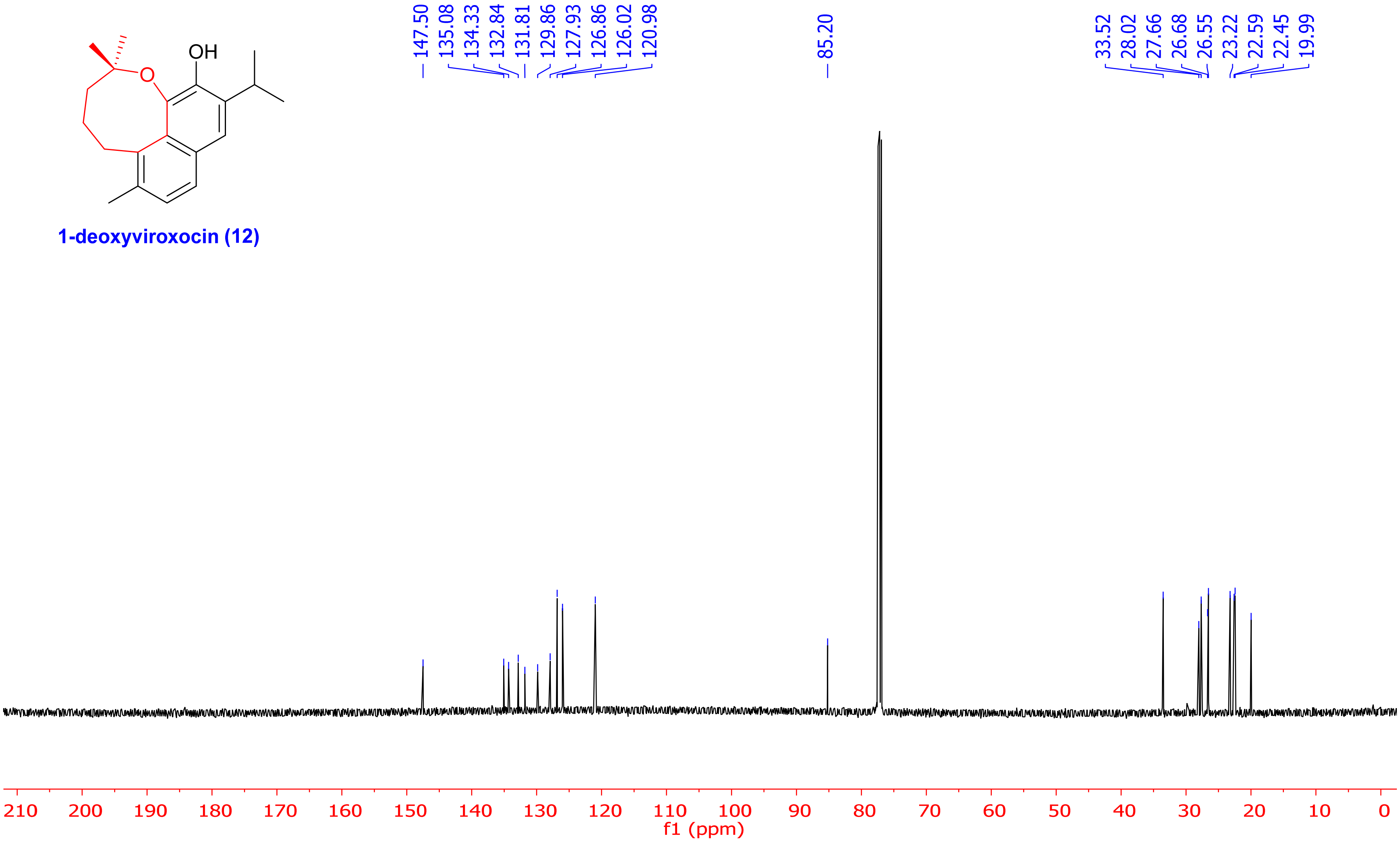

1-deoxyviroxocin (12)

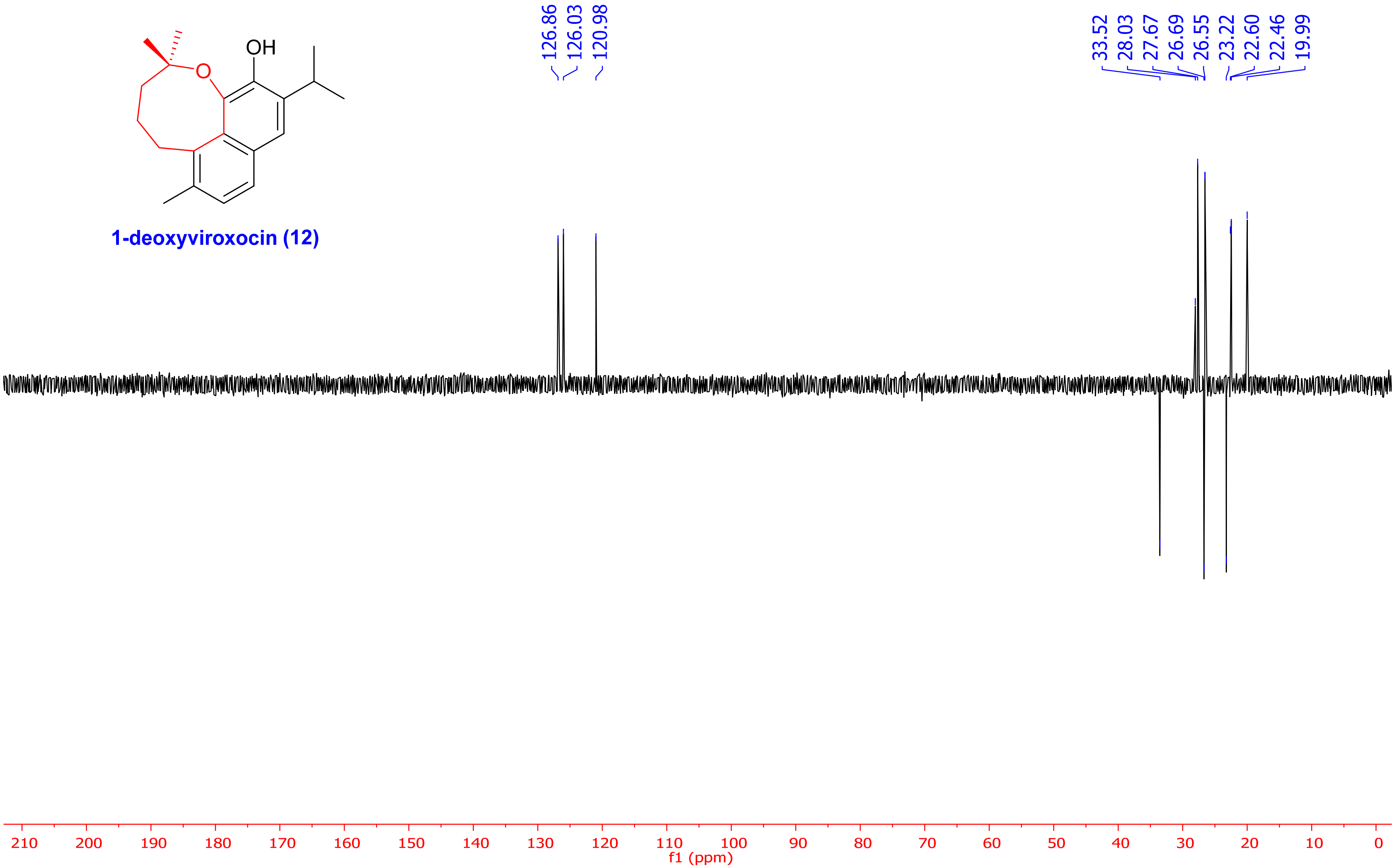
OH
O
1-deoxyviroxocin (12)
126.86
126.03
120.98
33.52
28.03
27.67
26.69
26.55
23.22
22.60
22.46
19.99
210
200
190
180
170
160
150
140
130
120
110
100
90
80
70
60
50
40
30
20
10
0
f1 (ppm)

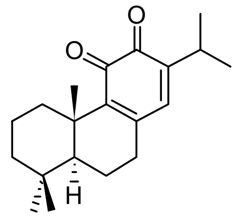

13

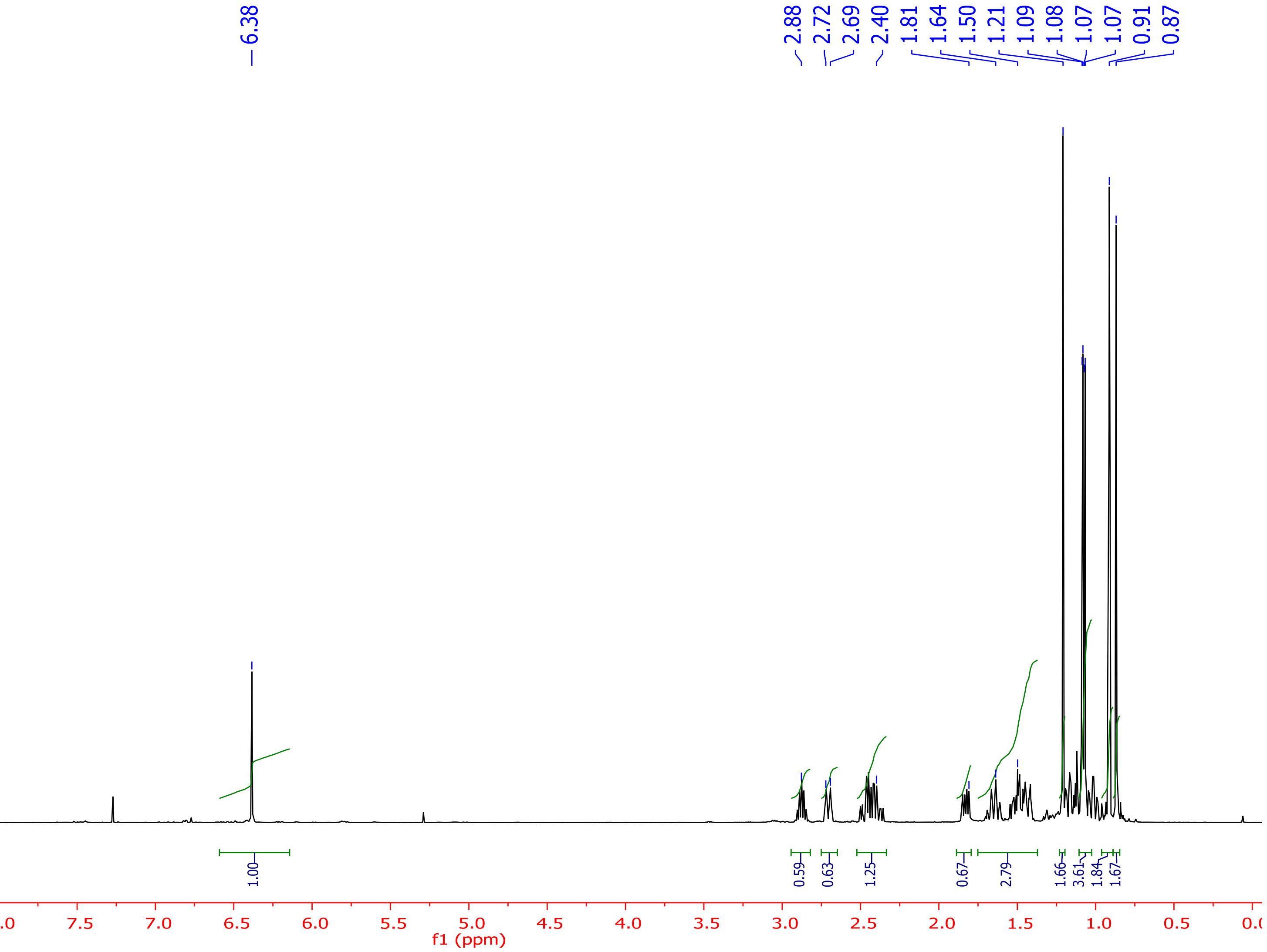

6.38
2.88
2.72
2.69
2.40
1.81
1.64
1.50
1.21
1.09
1.08
1.07
1.07
0.91
0.87
1.00
0.59
0.63
1.25
0.67
2.79
1.66
3.61
1.84
1.67
10.0 9.5 9.0 8.5 8.0 7.5 7.0 6.5 6.0 5.5 5.0 4.5 4.0 3.5 3.0 2.5 2.0 1.5 1.0 0.5
f1 (ppm)

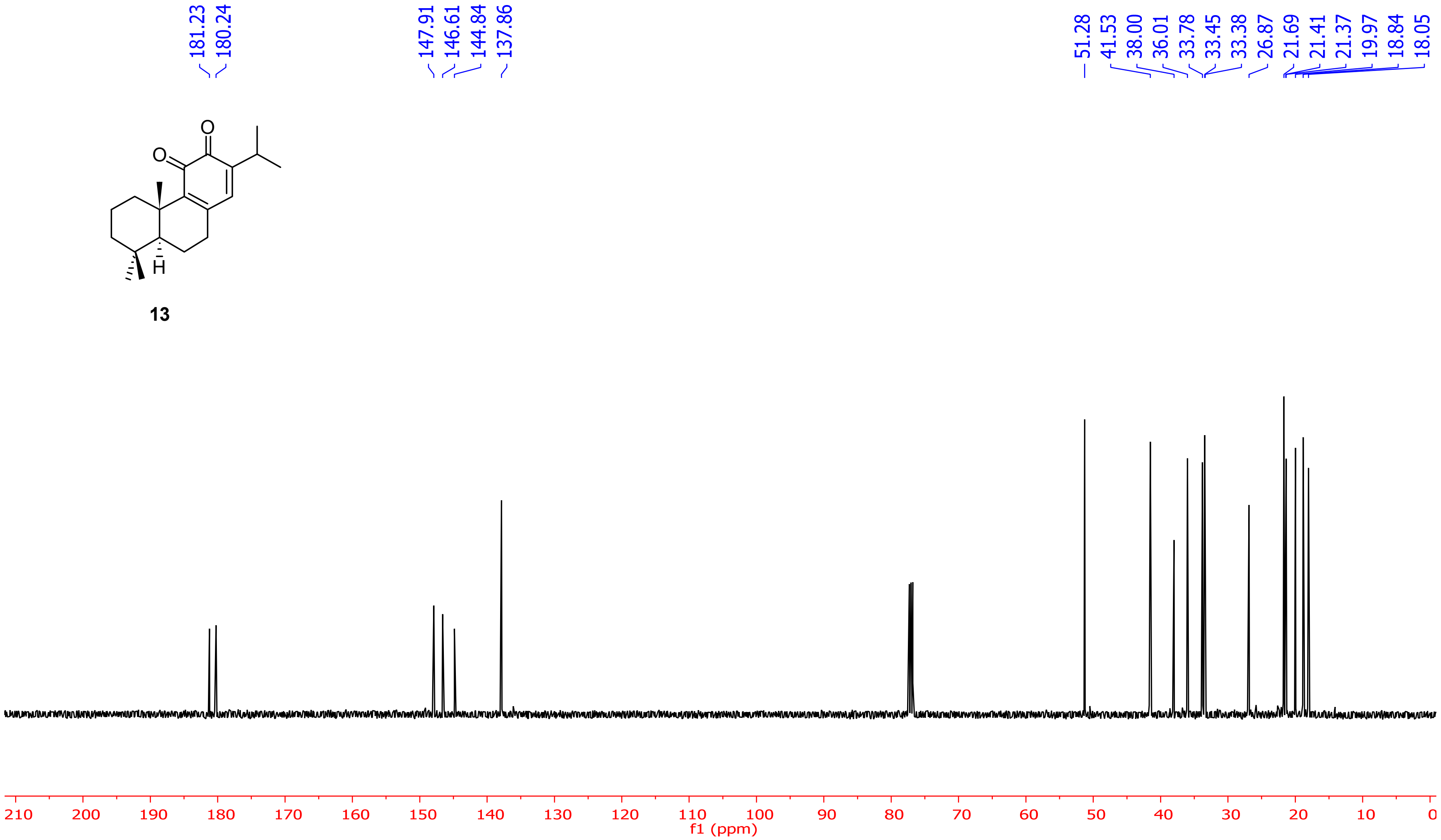
181.23
180.24
147.91
146.61
144.84
137.86
51.28
41.53
38.00
36.01
33.78
33.45
33.38
26.87
21.69
21.41
21.37
19.97
18.84
18.05
O
O
H
13
210 200 190 180 170 160 150 140 130 120 110 100 90 80 70 60 50 40 30 20 10 0
f1 (ppm)

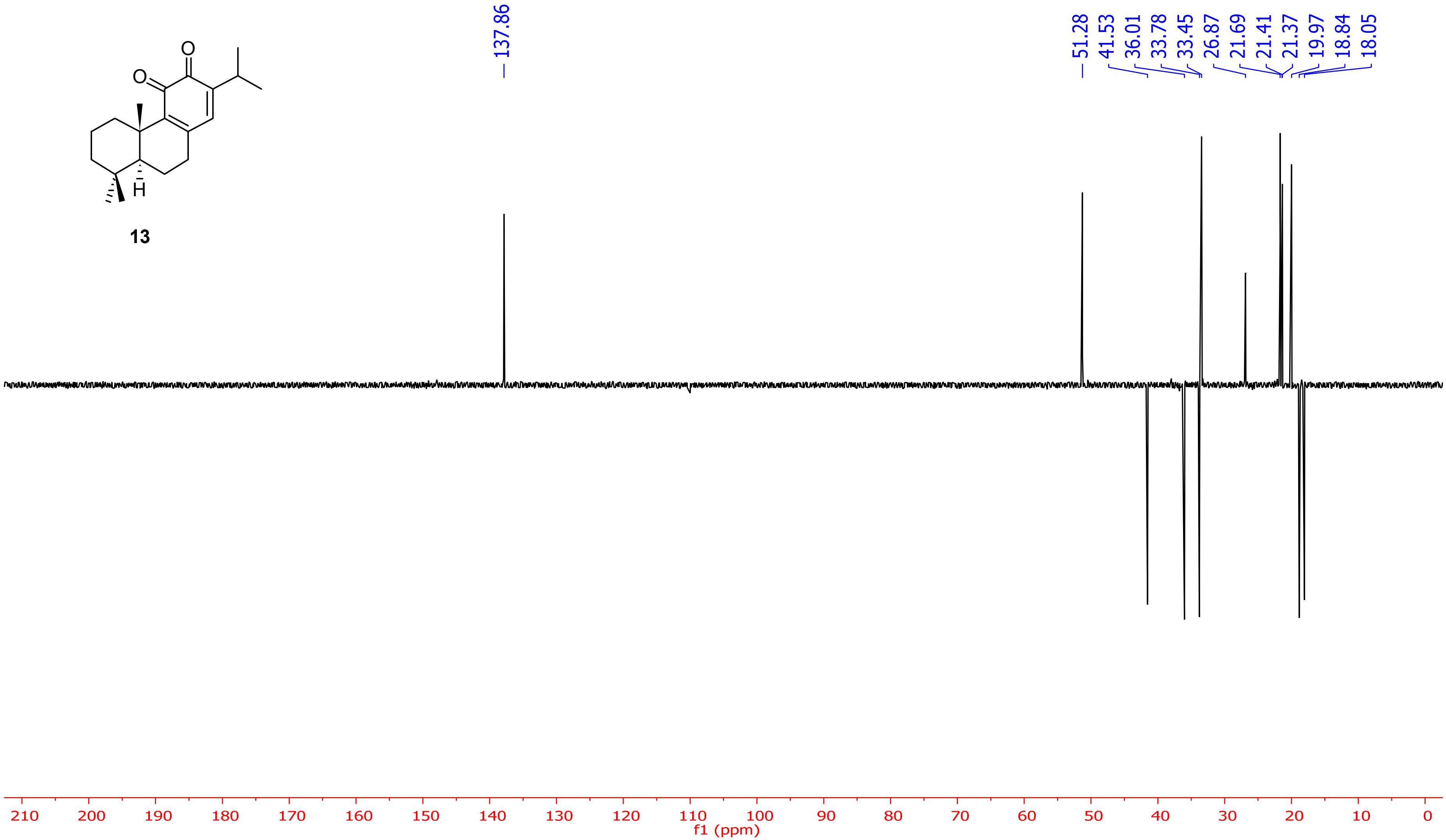
13
137.86
51.28
41.53
36.01
33.78
33.45
26.87
21.69
21.41
21.37
19.97
18.84
18.05
210
200
190
180
170
160
150
140
130
120
110
100
90
80
70
60
50
40
30
20
10
0
f1 (ppm)

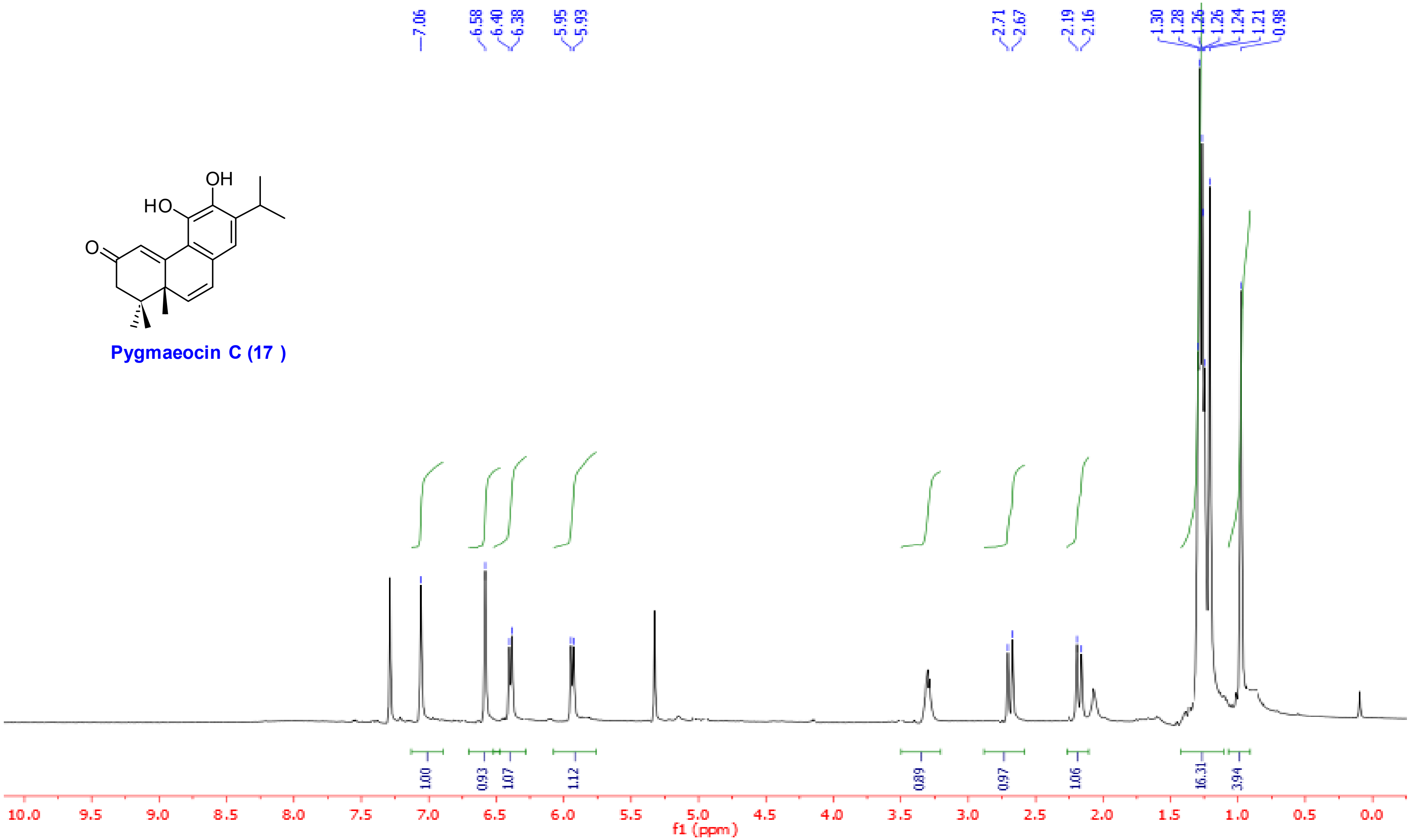

Pygmaeocin C (17)

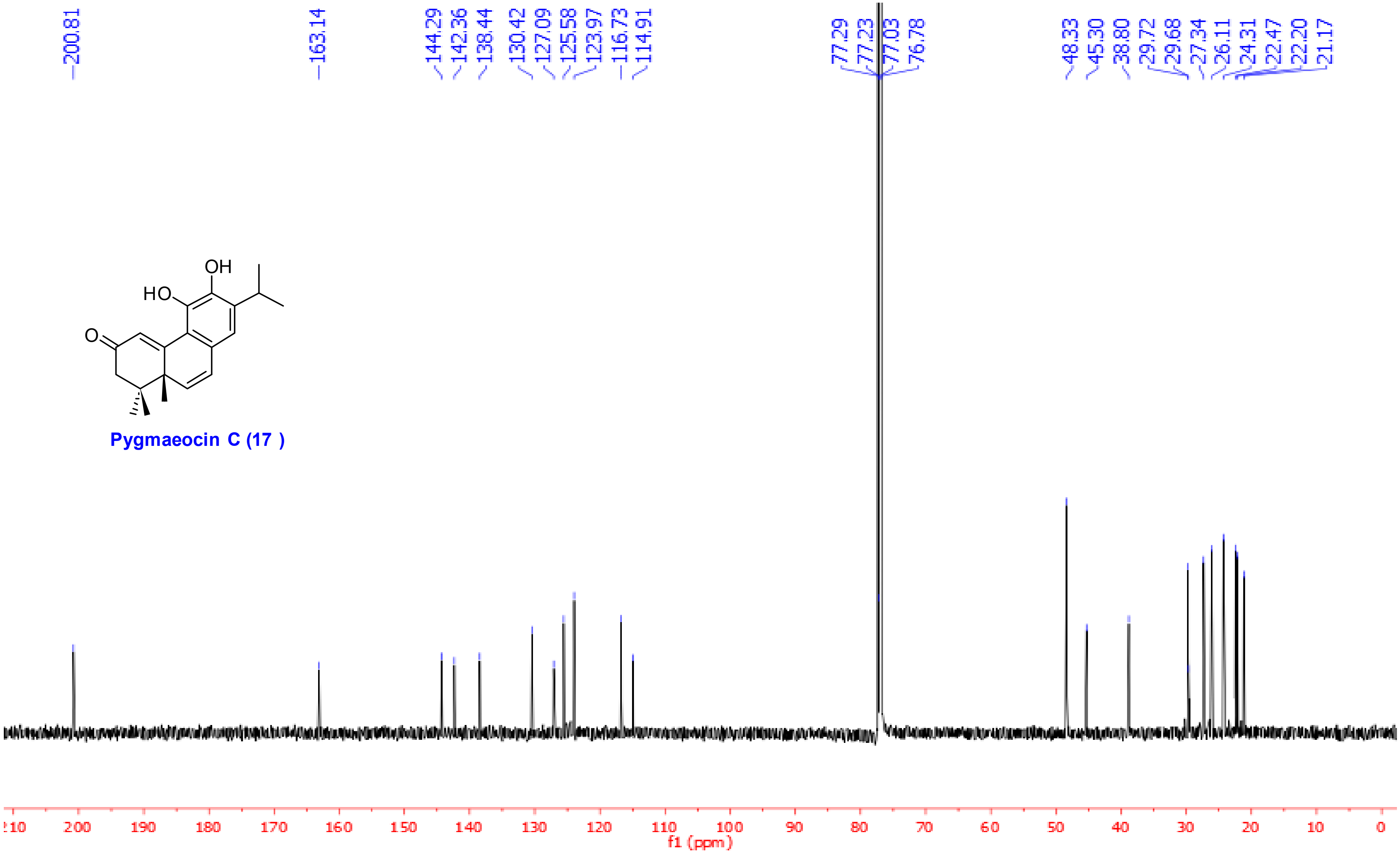
200.81
163.14
144.29
142.36
138.44
130.42
127.09
125.58
123.97
116.73
114.91
77.29
77.23
77.03
76.78
48.33
45.30
38.80
29.72
29.68
27.34
26.11
24.31
22.47
22.20
21.17
OH
HO
O
Pygmaeocin C (17)
210 200 190 180 170 160 150 140 130 120 110 100 90 80 70 60 50 40 30 20 10 0
f1 (ppm)

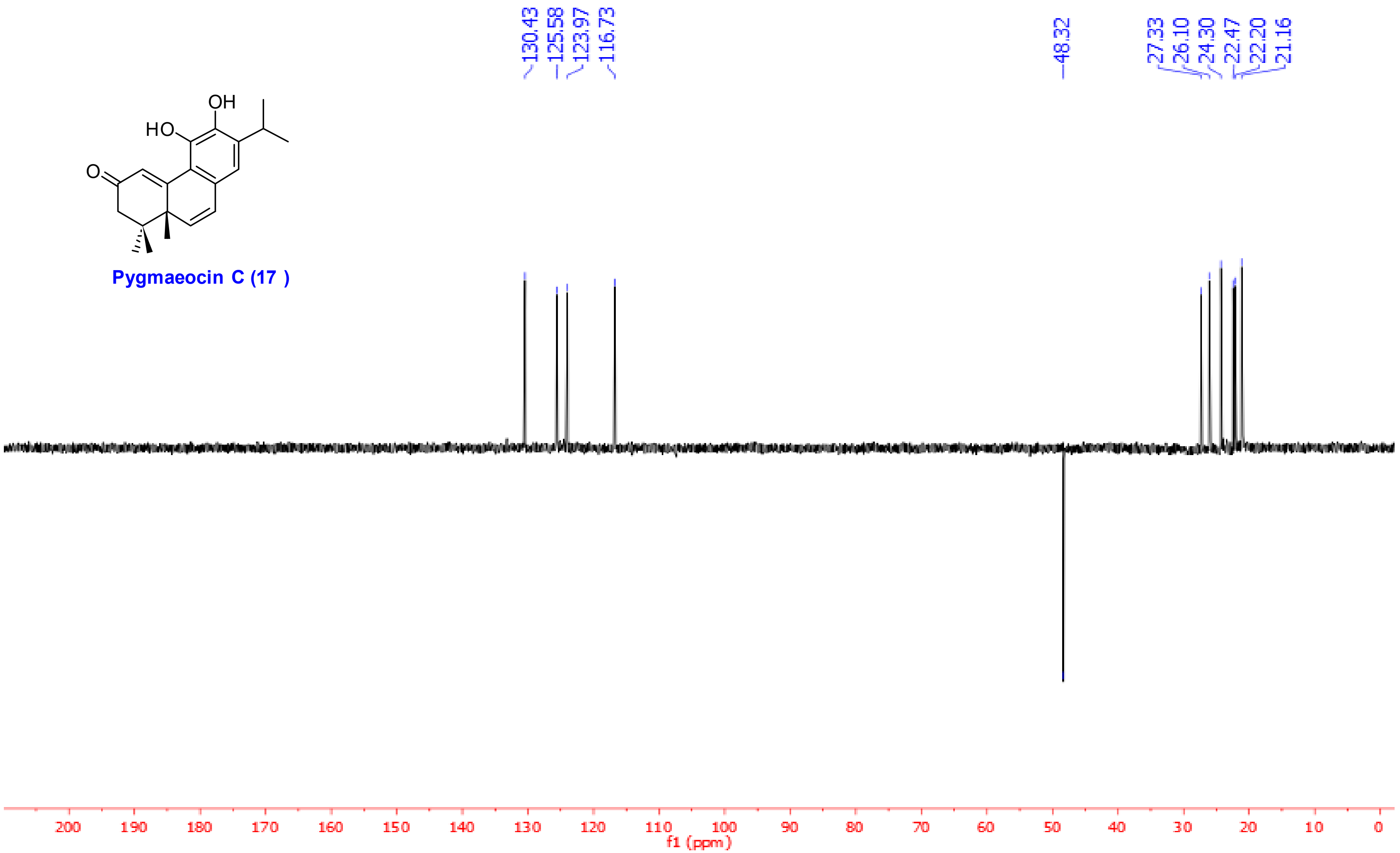

Pygmaeocin C (17)

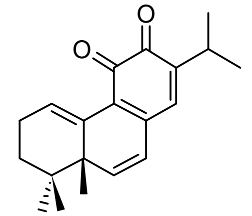

Viridoquinone (19)

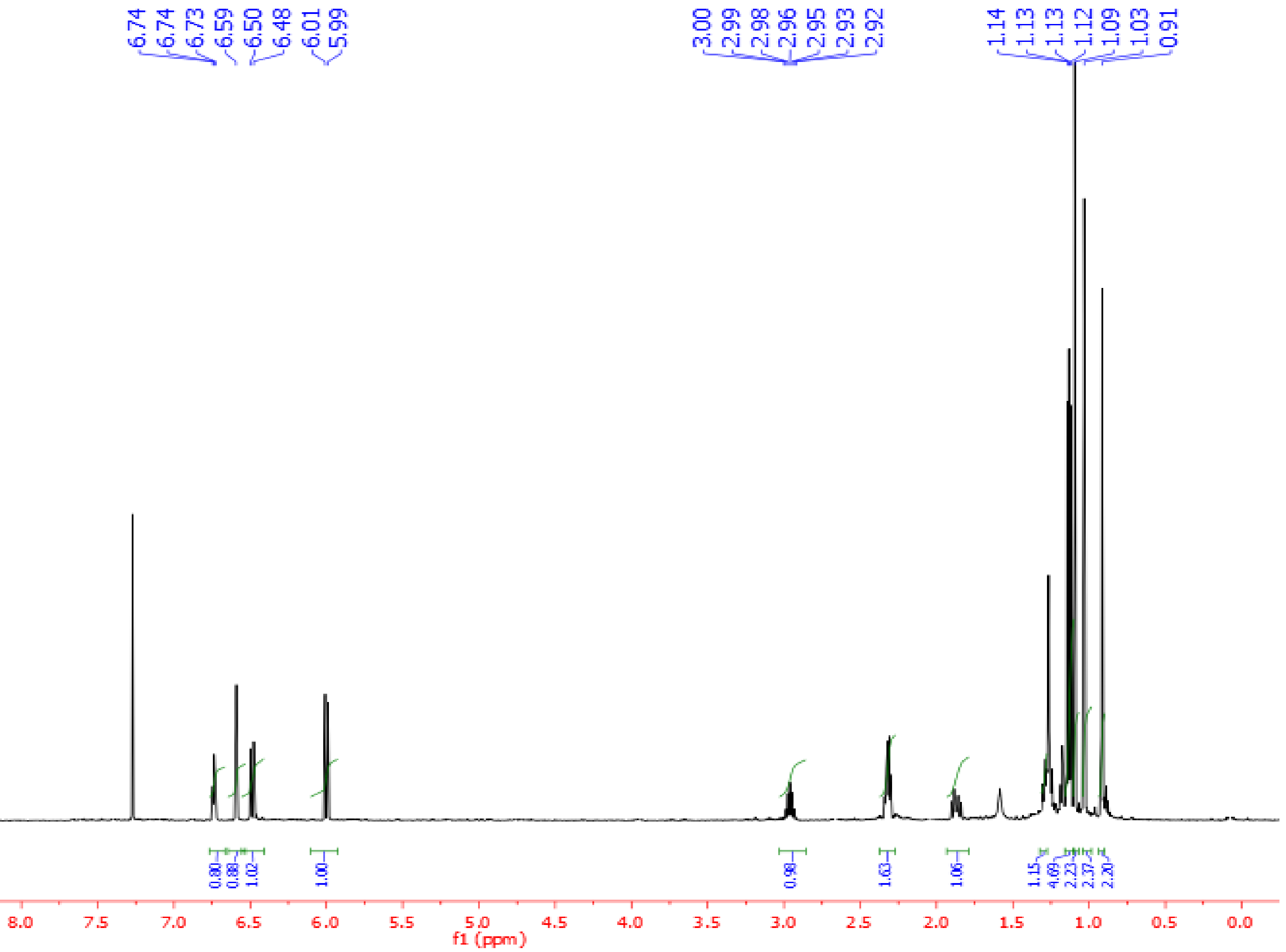

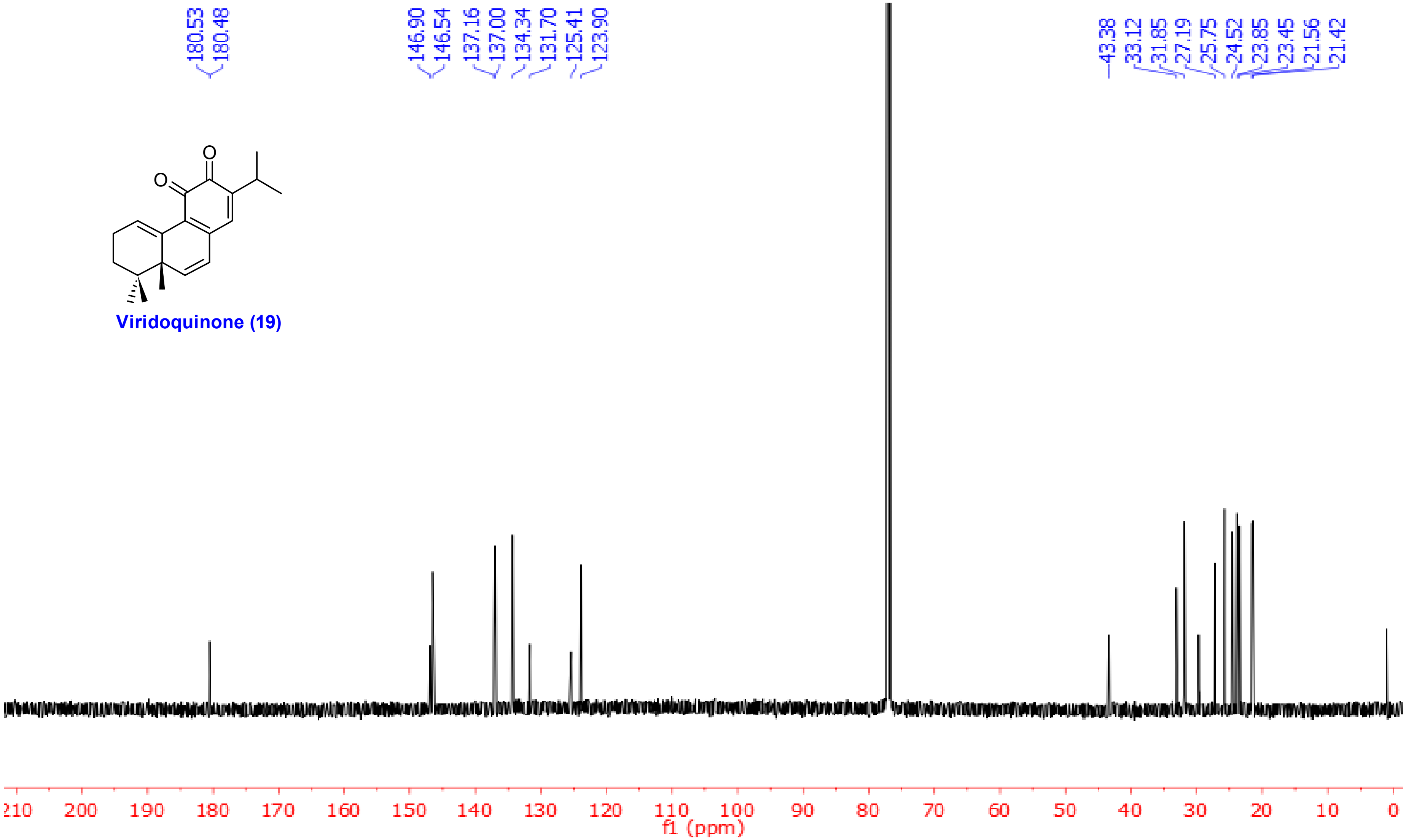
180.53
180.48
146.90
146.54
137.16
137.00
134.34
131.70
125.41
123.90
43.38
33.12
31.85
27.19
25.75
24.52
23.85
23.45
21.56
21.42
O
O
Viridoquinone (19)
210 200 190 180 170 160 150 140 130 120 110 100 90 80 70 60 50 40 30 20 10 0
f1 (ppm)

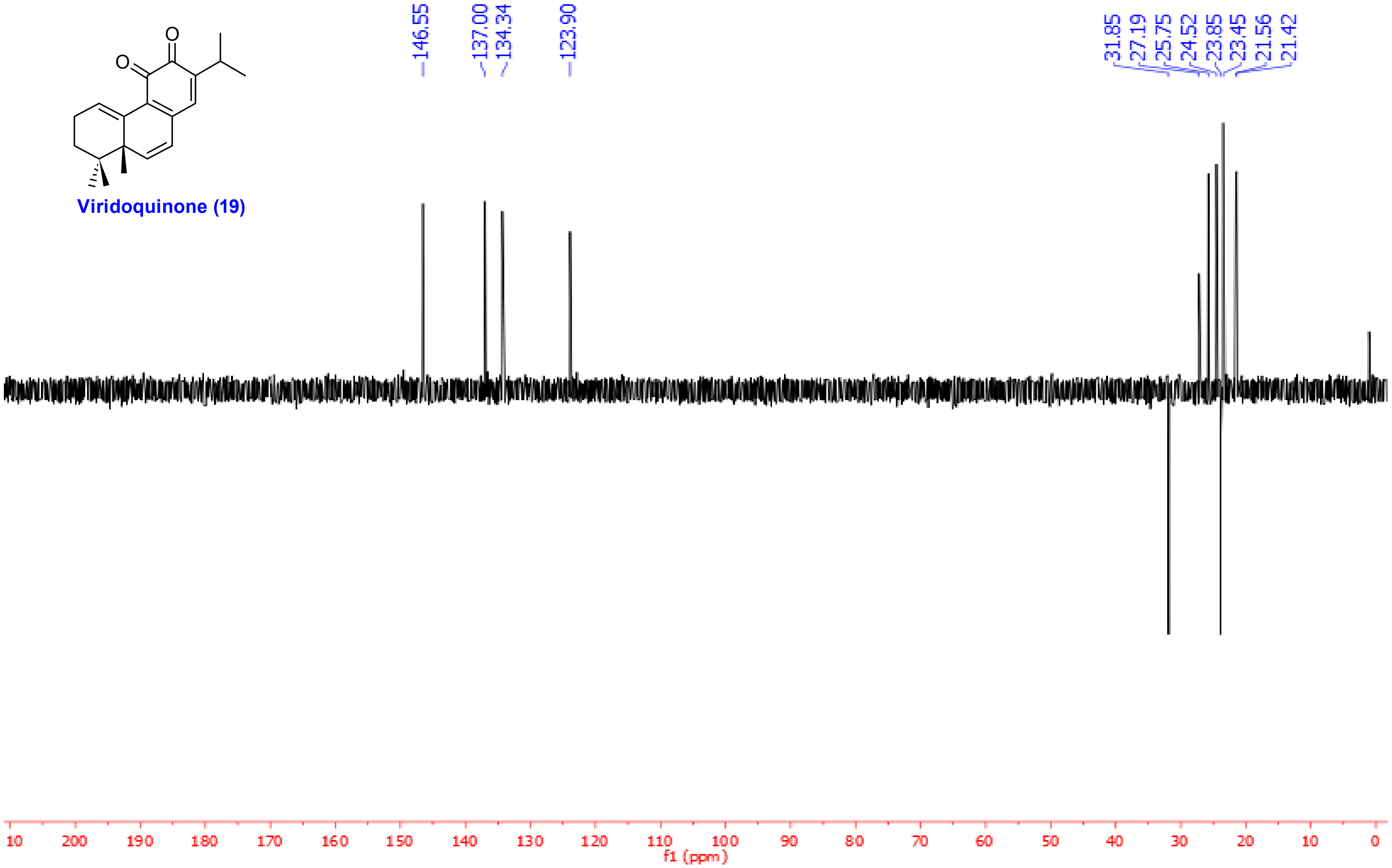

Viridoquinone (19)

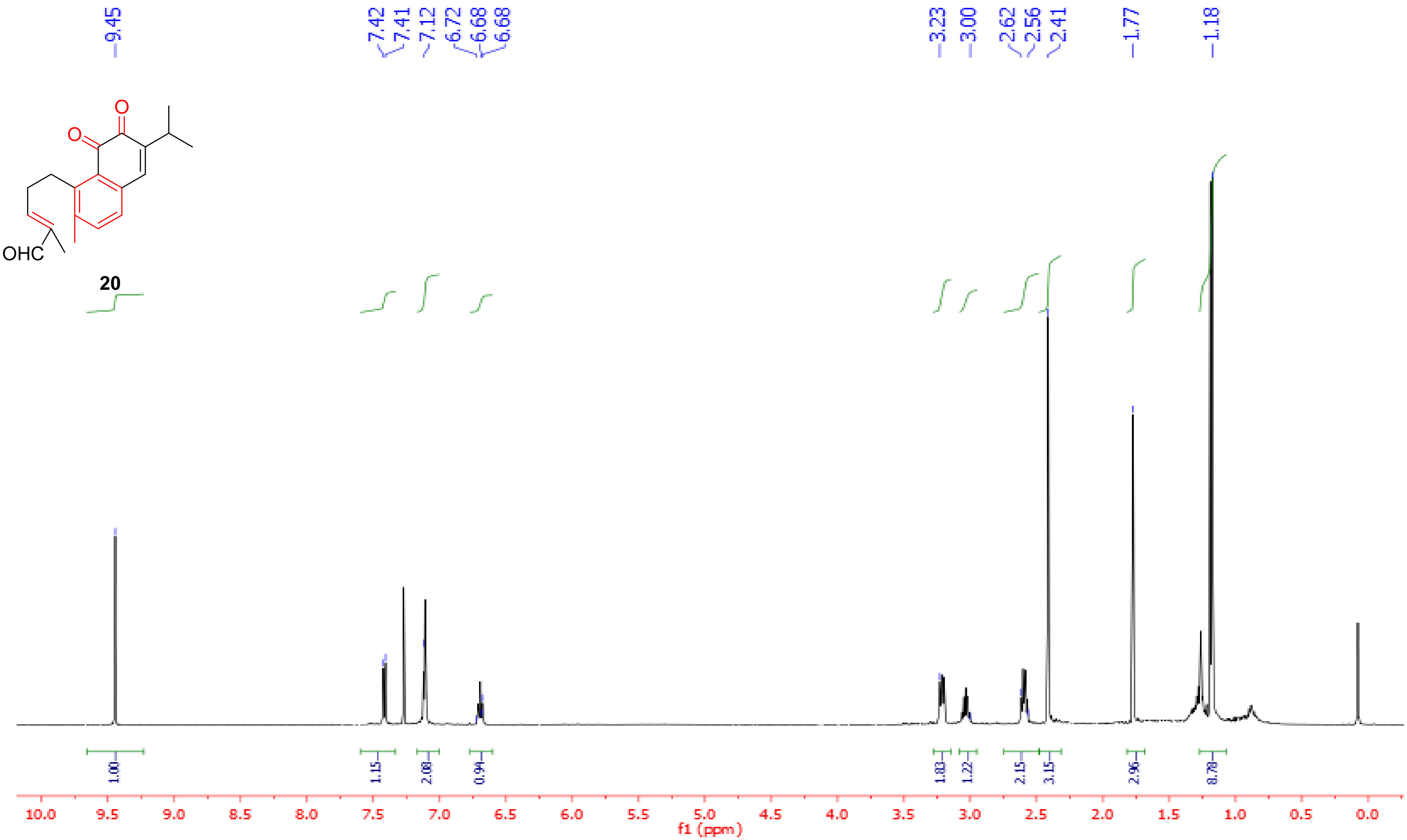
9.45
7.42
7.41
7.12
6.72
6.68
6.68
3.23
3.00
2.62
2.56
2.41
1.77
1.18
O
O
OHC
20
1.00
1.15
2.08
0.94
1.83
1.22
2.15
3.15
2.96
8.78
10.0 9.5 9.0 8.5 8.0 7.5 7.0 6.5 6.0 5.5 5.0 4.5 4.0 3.5 3.0 2.5 2.0 1.5 1.0 0.5 0.0
f1 (ppm)

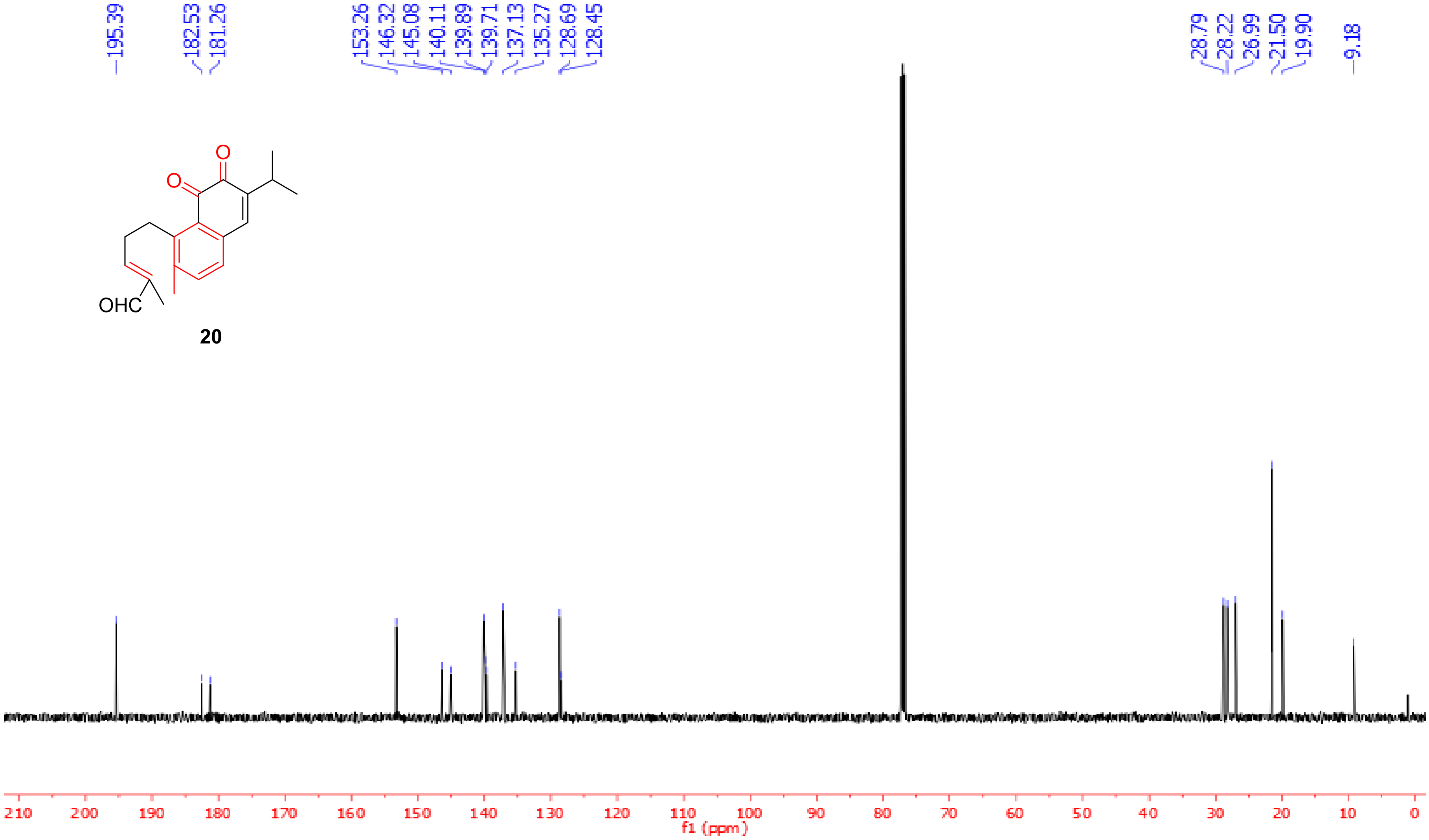

195.39
182.53
181.26
153.26
146.32
145.08
140.11
139.89
139.71
137.13
135.27
128.69
128.45
28.79
28.22
26.99
21.50
19.90
9.18
O
O
OHC
20
210 200 190 180 170 160 150 140 130 120 110 100 90 80 70 60 50 40 30 20 10 0
f1 (ppm)

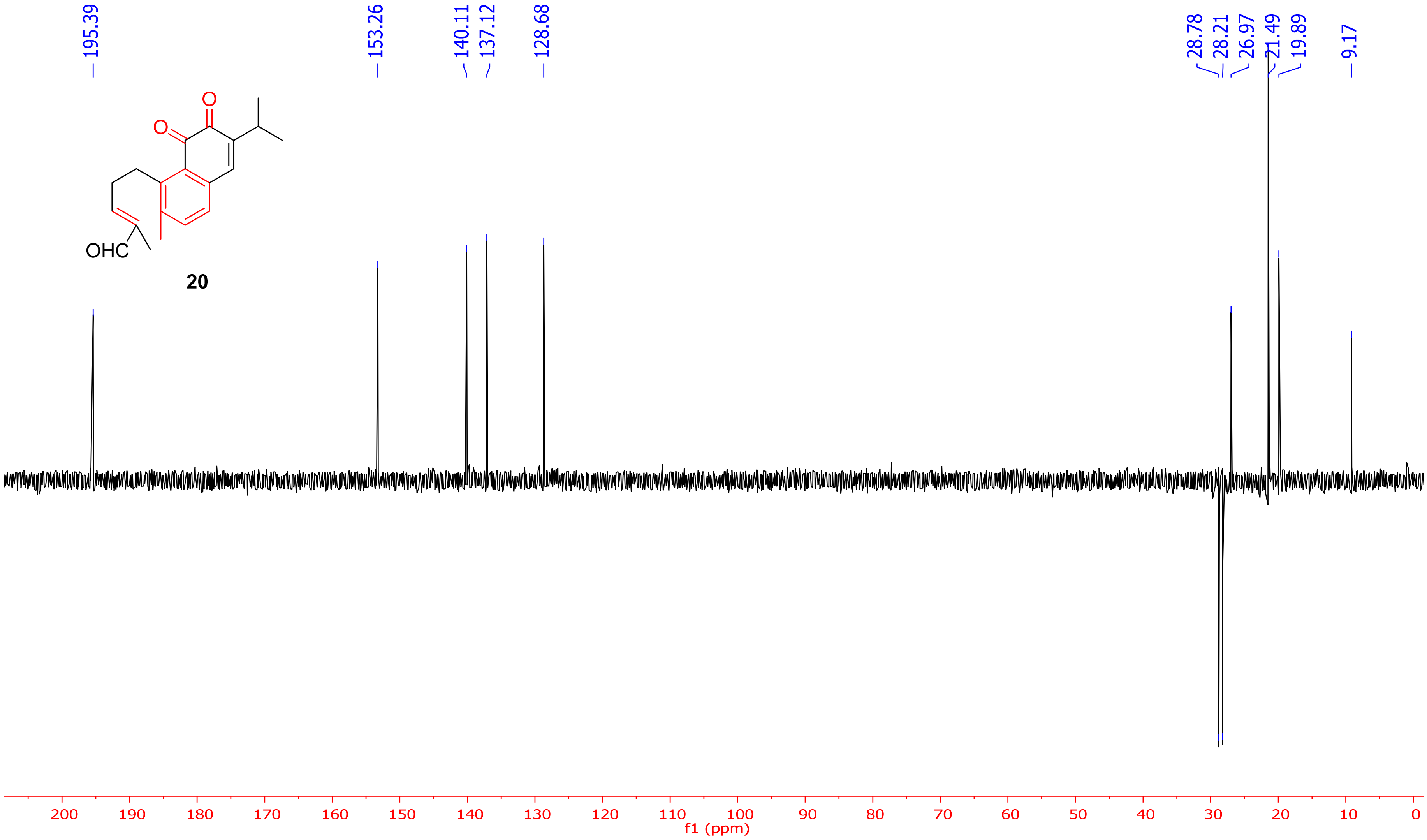
195.39
153.26
140.11
137.12
128.68
28.78
28.21
26.97
21.49
19.89
9.17
O
O
OHC
20
200
190
180
170
160
150
140
130
120
110
100
90
80
70
60
50
40
30
20
10
0
f1 (ppm)
